# Severe Propofol Infusion Syndrome Managed With Multimodal Critical Care: A Case Report

**DOI:** 10.7759/cureus.118902

**Published:** 2026-10-31

**Authors:** Rachael Power, Mohamed El Katan, Bradley Wibrow, Matthew H Anstey

**Affiliations:** 1 Anesthesiology Department, Tallaght University Hospital, Dublin, IRL; 2 Intensive Care Department, Sir Charles Gairdner Hospital, Perth, AUS; 3 Medical School, The University of Western Australia, Perth, AUS

**Keywords:** continuous renal replacement therapy (crrt), high-dose insulin euglycemic therapy, l-carnitine, propofol-related infusion syndrome, status epilepticus

## Abstract

Rapid cardiovascular collapse with severe metabolic derangement during propofol sedation should raise concern for propofol infusion syndrome (PRIS). A 21-year-old man with previous traumatic brain injury presented with refractory status epilepticus requiring mechanical ventilation and prolonged propofol sedation. Following four days of infusion, he developed abrupt bradycardia, cardiogenic shock, severe high-anion-gap metabolic acidosis, rhabdomyolysis, acute kidney injury, hypertriglyceridemia, and significant left ventricular dysfunction. The combination of prolonged propofol exposure and rapidly evolving multiorgan failure was considered consistent with PRIS. Propofol was discontinued immediately. Management included administration of multiple vasoactive infusions, sodium bicarbonate, calcium chloride, dual high-effluent continuous renal replacement therapy via two dialysis circuits, high-dose euglycemic insulin, and enteral L-carnitine. Hemodynamic improvement occurred within approximately six hours, followed by recovery of cardiac and renal function. He was discharged from the intensive care unit on day 30 and later returned home after rehabilitation without dialysis dependence. This case highlights the importance of early recognition of PRIS and rapid escalation of individualized organ support in severe presentations, while acknowledging that the contribution of individual adjunctive therapies cannot be determined from a single case.

## Introduction

Propofol infusion syndrome (PRIS) is an uncommon but frequently fatal complication of propofol exposure, particularly in critically ill patients receiving prolonged or escalating infusions. It is characterized by acute cardiovascular collapse accompanied by metabolic acidosis, rhabdomyolysis, renal failure, hyperkalemia, hypertriglyceridemia, and electrocardiographic abnormalities [1-3]. Diagnosis remains challenging because there are no universally accepted diagnostic criteria, and individual manifestations are nonspecific and may be attributed to sepsis, seizures, renal injury, or shock from other causes. Consequently, recognition relies on the clinical context and the evolution of a characteristic constellation of metabolic, cardiovascular, and muscular abnormalities. Reported risk factors include neurologic injury, refractory status epilepticus, critical illness, prolonged high-dose propofol infusion, concomitant catecholamine use, and possible impairment of mitochondrial fatty acid metabolism [2-4].

Mortality remains high, and management is not standardized beyond immediate discontinuation of propofol and aggressive supportive care. Although continuous renal replacement therapy (CRRT) may be used to manage severe metabolic derangement and acute kidney injury, evidence regarding the optimal intensity of extracorporeal support in PRIS is limited, and the role of simultaneous dual-machine CRRT has not been established. Similarly, high-dose euglycemic insulin has been proposed as a metabolic adjunct, but evidence supporting its use in PRIS remains limited. Neither strategy can therefore be regarded as an established treatment for PRIS.

We report severe PRIS in a young adult with refractory status epilepticus in whom simultaneous high-effluent dual-machine CRRT, high-dose euglycemic insulin, vasoactive support, and L-carnitine supplementation were used alongside immediate propofol cessation and supportive critical care. This case describes the diagnostic reasoning and practical use of these adjunctive strategies in rapidly progressive PRIS and adds to the limited clinical literature regarding dual-machine CRRT and high-dose insulin in its management, without establishing their efficacy.

## Case presentation

A 21-year-old man with a previous traumatic brain injury presented in refractory status epilepticus after an unwitnessed fall. He had sustained a severe traumatic brain injury several months previously requiring decompressive craniectomy and had recently returned home after inpatient rehabilitation. On presentation, he had ongoing seizure activity and required endotracheal intubation, mechanical ventilation, and admission to the intensive care unit. Seizures were treated with escalating-dose propofol infusion, fentanyl, and multiple antiepileptic agents. Continuous electroencephalography demonstrated persistent epileptiform activity, necessitating ongoing deep sedation. Propofol was continued for four days, with a mean infusion rate of 56.8 µg/kg/min. The infusion was initially increased from 27.4 to 57 µg/kg/min within the first two hours of ICU admission. Persistent seizure activity necessitated ongoing propofol sedation, with a final increase to 68.5 µg/kg/min for the 35 hours preceding clinical deterioration. The patient’s propofol exposure is summarized in Figure 1. 

**Figure 1:**
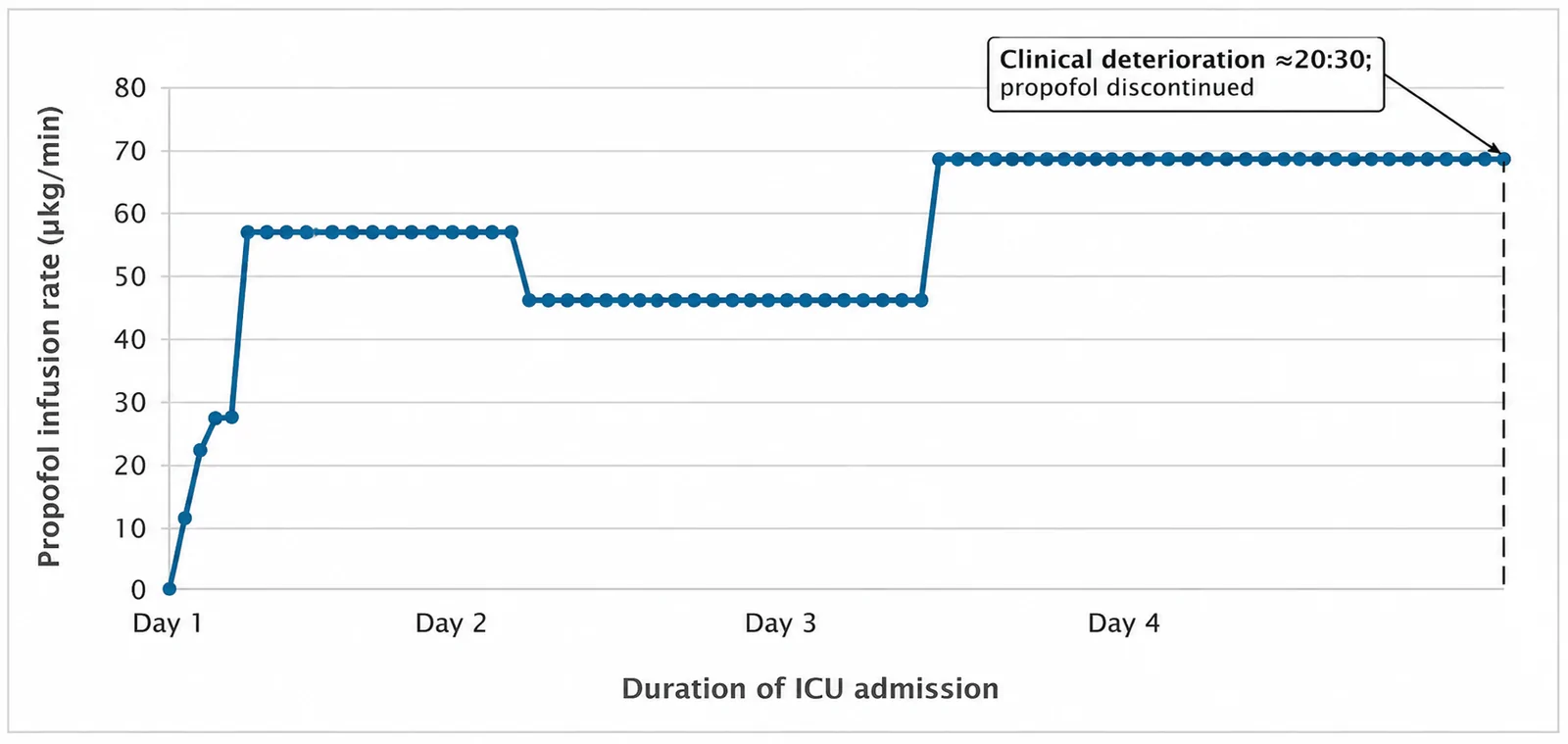
Propofol infusion rate during the four days of ICU admission preceding the development of propofol infusion syndrome Propofol was escalated during the ICU admission, with a final increase to 68.5 µg/kg/min for the 35 hours preceding acute clinical deterioration at approximately 20:30 on ICU day 4. Propofol was discontinued within approximately 5-10 minutes of the onset of hemodynamic instability. ICU: intensive care unit.

Diagnostic assessment

Metabolic abnormalities evolved during the ICU admission. At ICU admission, arterial pH was 7.25, bicarbonate 21 mmol/L, anion gap 16 mmol/L, and lactate 5.4 mmol/L. Lactate subsequently decreased to 1.6 mmol/L on the day preceding deterioration. On ICU day 4, at approximately 20:30, the patient deteriorated abruptly with profound bradycardia, rapidly progressive shock, pyrexia, and brown discoloration of the urine. Lactate increased to 10.3 mmol/L, accompanied by worsening high-anion-gap metabolic acidosis (pH 7.17, bicarbonate 12 mmol/L, anion gap 26 mmol/L).

At the time of acute deterioration on ICU day 4, creatine kinase was 62,600 IU/L (Table 1). Despite subsequent hemodynamic and metabolic improvement, creatine kinase continued to rise, reaching a peak of 931,000 IU/L two days later on ICU day 6. Other abnormalities at deterioration included hypertriglyceridemia (11.5 mmol/L), hyperkalemia (6.7 mmol/L), hyperphosphatemia, and rapidly progressive acute kidney injury, with creatinine increasing from 89 µmol/L at ICU admission to 497 µmol/L on the day of deterioration.

**Table 1:** Laboratory findings at ICU admission and day 4 deterioration Admission arterial blood gas (ABG) was obtained at 11:57 and the day 4 ABG at 22:24. ABG and laboratory measurements represent separate samples and should not be assumed to have been obtained simultaneously. ^†^CK was 62,600 IU/L at acute deterioration on ICU day 4 and subsequently peaked at 931,000 IU/L on ICU day 6. ^‡^Phosphate was not measured at ICU admission and was first measured on ICU day 2.

Parameter	ICU admission	Day 4 deterioration	Reference range
ABG pH	7.25	7.17	7.35-7.45
ABG pCO2 (mmHg)	49	34	36-45
ABG pO2 (mmHg)	105	162	85-110
ABG bicarbonate (mmol/L)	21	12	21-28
ABG base excess (mmol/L)	-5	-15	-3 to +3
ABG O2 saturation (%)	97	97	>95
ABG sodium (mmol/L)	142	145	135-145
ABG potassium (mmol/L)	3.9	6	3.5-5.2
ABG ionized calcium (mmol/L)	1.26	1.20	1.12-1.32
ABG chloride (mmol/L)	109	113	95-110
ABG anion gap (mmol/L)	16	26	7-17
ABG glucose (mmol/L)	7.6	14.3	3-5.4
ABG lactate (mmol/L)	5.4	10.3	<2.0
ABG hemoglobin (g/L)	159	139	115-160
ABG carboxyhemoglobin (%)	0.1	0.7	<6.0
ABG methemoglobin (%)	0.8	2.1	<1.5
Laboratory hemoglobin (g/L)	166	147	115-160
WBC (x10^9^/L)	7.43	26.99	4-11
Creatine kinase (IU/L)	Not measured on admission	62,600^†^	30-190
Urea (mmol/L)	4.6	11.2	3-8
Laboratory bicarbonate (mmol/L)	21	11	21-28
Creatinine (μmol/L)	89	497	60-110
eGFR (mL/min/1.73 m^2^)	>90	13	>90
Laboratory sodium (mmol/L)	141	143	135-145
Laboratory potassium (mmol/L)	4	6.7	3.5-5.2
Magnesium (mmol/L)	1.02	1.42	0.7-1.10
Phosphate (mmol/L)	Not measured on admission^‡^	3.11	0.75-1.50
Laboratory ionized calcium (mmol/L)	1.26	1.20	1.12-1.32
Triglycerides (mmol/L)	Not measured on admission	11.5	<2

Bedside transthoracic echocardiography demonstrated severe global left ventricular dysfunction with an estimated ejection fraction of 20%. Serial electrocardiograms demonstrated rapid progression from a near-normal tracing at 20:05 to axis and conduction abnormalities at 20:23 and severe conduction disturbance by 21:36, including QRS widening, T-wave abnormalities, and QTc prolongation to >500 ms (Figure 2).

**Figure 2:**
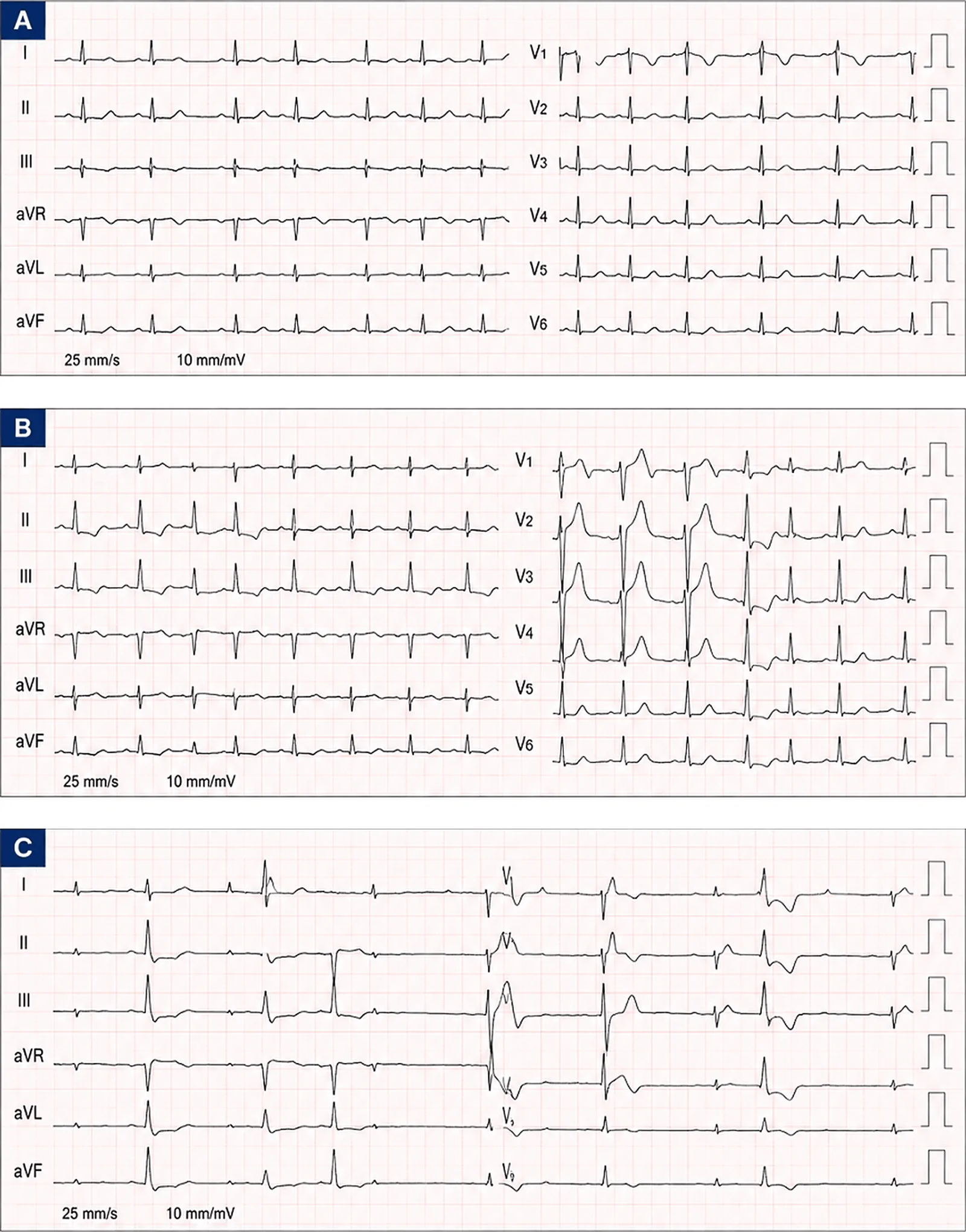
Serial 12-lead electrocardiograms demonstrating rapid progression of cardiac conduction abnormalities during propofol infusion syndrome (A) ECG at 20:05, before the onset of marked conduction disturbance. (B) ECG at 20:23, demonstrating evolving axis and conduction abnormalities. (C) ECG at 21:36, demonstrating severe conduction disturbance with marked QRS widening and repolarization abnormalities. During the deterioration, the corrected QT interval (QTc) exceeded 500 ms. Exact numerical QRS duration and uncorrected QT interval were not available from the source records. ECG calibration: 25 mm/s and 10 mm/mV.

Alternative explanations for the deterioration were considered. Persistent seizure activity was assessed with continuous electroencephalography and treated with multiple antiepileptic agents. Initial cerebral imaging demonstrated left-sided cerebral edema attributed to prolonged seizure activity. Infective central nervous system pathology was also considered early in the admission, and empirical ceftriaxone and acyclovir had been commenced. However, the abrupt development of cardiovascular collapse together with rapidly worsening lactic acidosis, severe rhabdomyolysis, hypertriglyceridemia, acute kidney injury, hyperkalemia, global left ventricular dysfunction, and evolving electrocardiographic abnormalities following prolonged and escalating propofol exposure raised strong clinical suspicion for PRIS. Alternative diagnoses were not considered to adequately explain the complete and rapidly evolving multi-system pattern.

Treatment

Propofol was discontinued within approximately 5-10 minutes of the onset of hemodynamic instability, and sedation was changed to midazolam and morphine. Minute ventilation was increased to compensate for the metabolic acidosis, and sodium bicarbonate and calcium chloride were administered. The mixed shock state required noradrenaline, adrenaline, dobutamine, and vasopressin.

A dialysis catheter was inserted within approximately one hour of deterioration and high-effluent CRRT was initiated. Dialysis flow and pre-pump replacement fluid were each increased to 2,000 mL/h, targeting an effluent rate greater than 25 mL/kg/h. Because of the severity of the metabolic acidosis, hyperkalemia, and rhabdomyolysis, a second dialysis catheter was subsequently inserted, permitting two CRRT machines to operate simultaneously to increase solute clearance and acid-base correction.

High-dose euglycemic insulin was initiated as an adjunct in the setting of profound myocardial dysfunction. Actrapid was administered as an initial 80-unit bolus followed by an infusion of 1 unit/kg/h (80 units/h), accompanied by 50% dextrose at 60 mL/h. Precise details of the glucose and electrolyte monitoring protocol and pre-specified treatment targets could not be reliably reconstructed from the available retrospective records and are therefore not reported. Enteral L-carnitine 500 mg three times daily was also commenced in the context of suspected mitochondrial dysfunction and concurrent valproate exposure [5,6].

Outcome and follow-up

Hemodynamic and metabolic improvement became apparent approximately six hours after propofol cessation and initiation of multimodal supportive therapy. Stabilization within hours of propofol cessation supported the working diagnosis and allowed avoidance of extracorporeal membrane oxygenation or plasma exchange. The patient continued to improve over the following two to three days, although creatine kinase continued to rise before reaching its peak of 931,000 IU/L on ICU day 6.

A tracheostomy was performed on day 18 because of prolonged ventilatory weaning associated with critical illness myopathy. He was discharged from the ICU on day 30 and required intermittent hemodialysis for a further two weeks. He was subsequently transferred for inpatient rehabilitation and ultimately returned home without ongoing dialysis requirements.

## Discussion

This case illustrates the importance of considering PRIS when a critically ill patient receiving propofol develops sudden cardiovascular collapse with otherwise unexplained metabolic, renal, and muscular injury. Several features increased the likelihood of PRIS in this patient, including refractory status epilepticus, previous neurologic injury, prolonged propofol exposure, catecholamine-requiring shock, hypertriglyceridemia, rhabdomyolysis, and severe left ventricular dysfunction. Although no universally accepted diagnostic criteria exist, the temporal association with propofol and the simultaneous development of classic clinical and biochemical abnormalities supported the diagnosis [1-4].

The cornerstone of management is immediate cessation of propofol. However, patients with severe PRIS often require rapid escalation of organ support while alternative sedative and antiseizure strategies are instituted. In this case, severe metabolic acidosis, hyperkalemia, rhabdomyolysis, and acute kidney injury were treated with high-effluent CRRT. A second dialysis catheter and dual-machine CRRT were used because the severity of the metabolic derangement required rapid correction; reports describing this approach are limited [7]. High-dose euglycemic insulin was used to support myocardial carbohydrate utilization and contractility during profound cardiogenic shock. Although best described in toxicologic cardiogenic shock, its role in PRIS is not well defined [8]. L-Carnitine was given because propofol-related mitochondrial dysfunction and concurrent valproate therapy may impair fatty acid metabolism and contribute to acquired carnitine deficiency [5,6].

Other rescue therapies described in severe PRIS include therapeutic plasma exchange and extracorporeal circulatory support, particularly when refractory shock or severe hypertriglyceridemia persists despite conventional therapy [9,10]. These were considered but not required, as the patient improved after propofol cessation and multimodal supportive management.

The favorable outcome in this case demonstrates the feasibility of incorporating advanced organ support within a multimodal management strategy for severe PRIS. However, because several interventions were introduced in close succession, the relative contribution of dual-machine CRRT, high-dose insulin, L-carnitine, and other supportive measures cannot be determined. This case therefore illustrates a practical multimodal approach rather than the effect of any single therapy, while reinforcing the importance of prompt recognition and escalation.

​​​For general physicians and trainees, the case also emphasizes communication and systems-based practice. PRIS is rare enough that few clinicians will have personal experience with it, yet delays in stopping propofol may be catastrophic. A shared mental model between intensivists, neurologists, anesthesiologists, nurses, renal physicians, and perfusion or extracorporeal teams can shorten the interval between recognition and definitive supportive therapy.

## Conclusions

Clinicians should consider propofol infusion syndrome when a patient receiving propofol develops otherwise unexplained cardiovascular collapse accompanied by metabolic, renal, or muscular injury. Propofol should be discontinued promptly when the syndrome is suspected, while alternative sedation and supportive treatment are instituted. Severe presentations may require rapid, coordinated escalation of renal and cardiovascular organ support. This case demonstrates the feasibility of a multimodal, multidisciplinary management strategy and was associated with a favorable outcome, while the contribution of individual adjunctive therapies remains uncertain.
